# No effect of cognitive behavioral patient education for patients with pain catastrophizing before total knee arthroplasty: a randomized controlled trial

**DOI:** 10.1080/17453674.2019.1694312

**Published:** 2019-11-25

**Authors:** Sara Birch, Maiken Stilling, Inger Mechlenburg, Torben Baek Hansen

**Affiliations:** a Department of Physiotherapy and Occupational Therapy, Holstebro Regional Hospital, Hospital Unit West;; b Department of Clinical Medicine, Aarhus University;; c University Clinic for Hand, Hip, and Knee Surgery, Holstebro Regional Hospital, Hospital Unit West;; d Department of Orthopaedic Surgery, Aarhus University Hospital;; e Department of Public Health, Aarhus University, Denmark

## Abstract

Background and purpose — Pain catastrophizing contributes to acute and long-term pain after total knee arthroplasty (TKA) but currently there are only limited treatment options. This study investigates the effectiveness of patient education in pain coping among patients with moderate to high pain catastrophizing score before TKA. Secondary outcomes were physical function, quality of life, self-efficacy, and pain catastrophizing.

Patients and methods — The study was a parallel-group randomized controlled trial including patients with moderate to high levels of pain catastrophizing. 60 patients were recruited from December 2015 to June 2018. The mean age of the patients was 66 (47–82) years and 40 were women. The patients were randomized to either cognitive-behavioral therapy (CBT) based pain education or usual care. The primary outcome measure was pain under activity measured with the Visual Analog Scale (VAS). All outcomes were measured preoperatively, at 3 months, and at 1 year after surgery.

Results — We found no difference in the primary outcome measure, VAS during activity, between the 2 groups but both groups had large reductions over time. The CBT-based pain education group reduced their VAS score by 37 mm (95% CI 27–46) and the control group by 40 mm (CI 31–49). We found no statistically significantly differences between the 2 groups in any of the secondary outcomes.

Interpretation — Future research is warranted to identify predictors of persistent pain and interventions for the approximately 20% of patients with persisting pain after a TKA.

Up to 20% of patients report persistent pain after a total knee arthroplasty (TKA) (Beswick et al. 2012). Both physical and psychological risk factors for poor outcome after TKA are identified, highlightning the complex interaction between physiological, biological, and social factors. Psychological factors such as anxiety, self-efficacy, kinesiophobia, and pain catastrophizing are associated with persistent pain after TKA (Hirakawa et al. 2014, Bierke et al. 2017). Pain catastrophizing is defined as negative emotional and cognitive responses to actual or anticipated pain (Sullivan et al. 1995). A Cochrane Review has shown that cognitive behavioral therapy (CBT) is an effective treatment for many different chronic pain conditions (Williams et al. 2012) and interventions such as pain coping skills training based on CBT are shown to reduce pain catastrophizing and improve knee function for patients with knee osteoarthritis (OA) (Somers et al. 2012, Broderick et al. 2014, Bennell et al. 2016, Cai et al. 2018).

However, only 1 recent study has examined a cognitive behavioral intervention for patients with knee OA and high levels of pain catastrophizing. Riddle et al. (2019) studied 402 patients in a 3-armed randomized clinical trial (pain coping, arthritis education, and usual care) and found no differences in any of the outcomes between the 3 treatment arms.

The primary aim of this study was to investigate the effectiveness – on pain after TKA – of pre- and postoperative patient education in pain coping among patients with moderate to high pain catastrophizing score. Further, we wanted to investigate the effectiveness on postoperative physical function, quality of life, self-efficacy, and pain catastrophizing.

## Patients and methods

### Participants

A protocol article describes the patients and methods in detail (Birch et al. 2017).

This randomized controlled trial recruited patients from the orthopedic outpatient clinic at Holstebro Regional Hospital from December 2015 to June 2018. Inclusion criteria were primary knee OA, scheduled for primary total knee arthroplasty because of osteoarthritis, age ≥ 18, a score > 22 on the Pain Catastrophizing Scale (PCS), proficiency in written and spoken Danish, and informed written consent.

The patients were excluded if they had severe depression as diagnosed with the Major Depression Inventory (MDI) or were planning to have a contralateral knee arthroplasty within 1 year after the operation.

The patients were included consecutively and randomized 1:1 to either the intervention group or usual care in 7 blocks of 8 persons. Because of high withdrawal rate, we decided to continue including patients and randomized another 16 patients. At the predefined deadline of June 1, 2018 we stopped the inclusion of patients. The physiotherapists and nurses who did the baseline and follow-up testing where blinded for the randomization. It was not possible to blind the patients and the 2 physiotherapists who delivered the patient education.

### Control and intervention group

Patients in the control group received usual care. Preoperatively this consisted of a multidisciplinary information meeting for patients and their relatives. Postoperative patients in the usual care group were offered a phone call after 1 week and 3 control visits at the hospital after 2, 4, and 12 weeks.

In addition to usual care the intervention group participated in patient education based on CBT. The patient education consisted of 7 individual sessions of approximately 45 minutes, 3 sessions preoperatively and 4 sessions postoperatively. The 1st session was delivered approximately 2 weeks before the operation and the last session was given 3 months after the operation. The patient education was delivered by 2 physiotherapists who followed a manual describing in detail the content of each of the 7 sessions. This ensured that each session was standardized although there was room to discuss individual needs. The physiotherapists observed each other on a regular basis to make sure that they delivered the intervention in the same way. As far as possible the same physiotherapist followed the patient through all the sessions. The patient education covered 3 main components: (1) education in pain and the interaction between cognition and pain perception; (2) training in cognitive and behavioral pain coping skills; and (3) training in how to apply the learned coping skills in real-life situations. For details see the protocol (Birch et al. 2017).

### Deviations from protocol

In November 2016, approximately 1 year after we started including patients, we had to change the number of sessions in the patient education from 7 to 6. The reason for this was that we had to exclude too many patients because the waiting time between the outpatient surgeon consultation and the operation was too short to deliver the 3 preoperative sessions. Thus, we decided to combine the 3 sessions before the operation into 2, but without changing the content.

### Outcome measures and follow-up

The primary outcome measure was pain during activity at 12 months measured with the Visual Analog Scale (VAS): 0 indicating “no pain,” to 100 indicating “worst pain imaginable” on a mm scale. Pain during activity was measured right after the 6-minute walk test. Secondary outcomes included physical function, quality of life, physiological factors, and pain during rest. The Oxford Knee Score (OKS) was used to measure knee-related pain and physical function. Overall scores run from 0 to 48 with 48 being the best outcome (Paulsen et al. 2012). 2 performance-based outcomes—the 6-minute walk test and sit to stand in 30 seconds—were used to measure physical function (Enright 2003, Gill and McBurney 2008). The Pain Catastrophizing Scale (PCS) was used to address feelings and thoughts related to pain. Total score ranges from 0 to 52. The higher the score, the more catastrophizing thoughts are present (Sullivan et al. 1995). The EQ-5D is a measure of self-reported general health and consists of 5 dimensions: mobility, self-care, usual activities, pain/discomfort, and anxiety/depression. The Pain Self-Efficacy Questionnaire (PSEQ) measures the patient’s beliefs in his/her ability to perform activities despite pain. The score ranges from 0 to 60, with 60 being the best outcome (Rasmussen et al. 2016). Pain at rest was measured with VAS before the 2 performance-based tests. The Knee Injury and Osteoarthritis Outcome Score (KOOS) consists of 5 subscales. We used the subscale “pain.” After calculation the score ranges from 0 to 100, where 100 is indicating no symptoms (Roos et al. 1998). All outcomes were measured preoperatively, at 3 months, and at 1 year after surgery.

### Sample size

VAS during activity was used to calculate the needed study sample. Based on former studies we used a minimal clinically important difference in VAS activity of 18 mm (Hagg et al. 2003) between the 2 groups and a standard deviation of 19 mm (Forsythe et al. 2008, Edwards et al. 2009, Papakostidou et al. 2012). With a significance level at 0.05, a power of 90%, and an expected loss to follow-up of approximately 20% of the patients, a total sample of 56 patients was needed.

### Statistics

Normally distributed data are described by mean (SD), and data not normally distributed by median (range). In the analysis the intention-to-treat principle was used including all randomized participants. For all outcomes, between-group mean differences, changes over time, and 95% confidence intervals (CI) from baseline to 3 and 12 months were analyzed by a mixed-effects linear regression model with a random person level and systematic effects of time, group, and the interaction between time and group. In the analysis of change in the primary outcome from baseline to 12 months we included VAS during activity at baseline as a covariate. Model validation was performed by comparing observed and expected within-subject standard deviations and correlations and by inspecting QQ plots. We tried to include morphine intake as a covariate, but this did not change the results or conclusions.

We filled missing values with mean values as described in the manuals if less than half of the answers were missing in the SF-36 (PF) and if 2 or fewer of the answers were missing in the OKS, the PSEQ, and the PCS. In each questionnaire missing values were filled with mean values between 0 and 5 times.

The significance level was set at < 0.05. The statistical analyses were performed using STATA 15 (StataCorp, College Station, TX, USA) software package.

### Ethics, registration, data sharing, funding, and potential conflict of interests

The study was conducted in accordance with the Declaration of Helsinki and the CONSORT statement, and registered in the Danish Data Protection Agency (j.nr. 1-16-02-245-15). All patients gave informed written consent. The protocol was approved by the Central Denmark Regions Committee on Biomedical Research Ethics (journalno. 1-10-72-64-15, issue date March 25, 2015) and registered at ClinicalTrials.gov (NCT02587429). The study was supported by the Tryg Foundation (grant number: 113944) and the Danish Rheumatism Association (grant number: A3622). None of the funders have any role in the study other than providing funding. The authors have no competing interests. Data is available from the corresponding author on reasonable request.

## Results

### Patient flow

**Figure 1.**
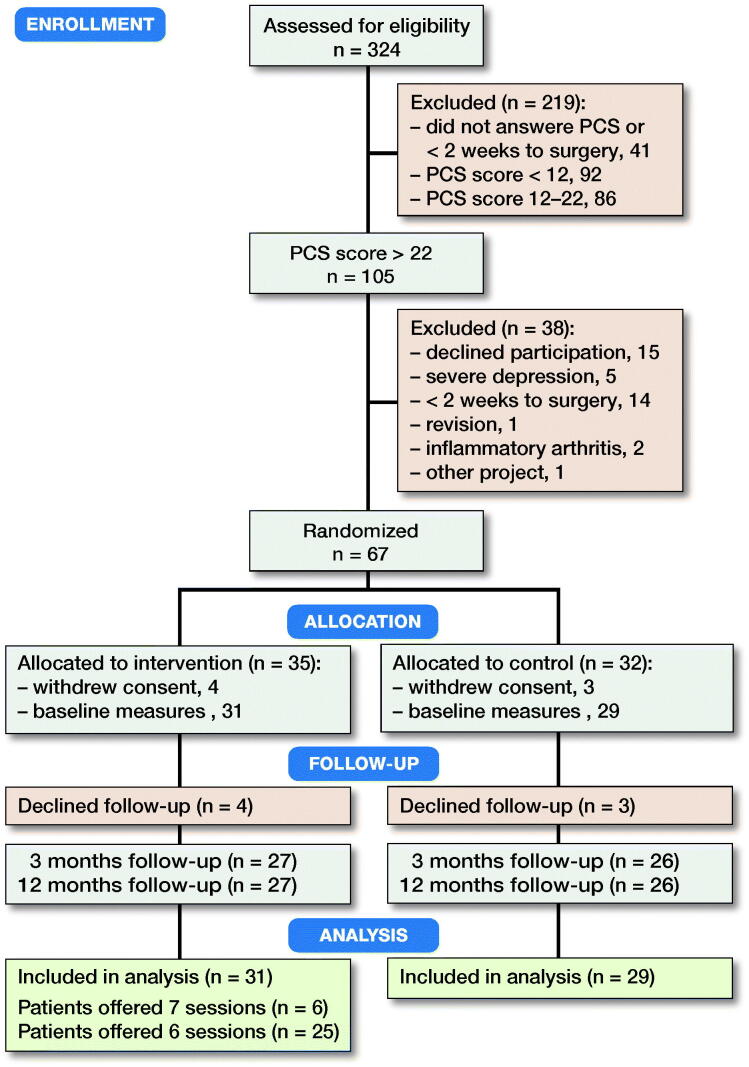
CONSORT flowchart of the trial. PCS = Pain Catastrophizing Scale

From December 2015 to June 2018, 324 patients were considered for inclusion. 283 patients were screened and 105 of these had a PCS score > 22. We randomized 67 patients but shortly after the randomization 7 patients decided not to participate and 60 patients were measured at baseline and included in the analysis (Figure 1). Of the 31 patients randomized to receive the patient education 6 patients received 7 sessions and 25 patients received 6 sessions because of the described change in protocol. The compliance with the intervention was high and only 2 of the 31 patients missed 1 session due to personal circumstances. The 2 groups were similar at baseline (Table 1), except that a substantially higher number of the patients in the pain education group used opioids or morphine.

**Table 1.** Baseline demographics and characteristics. Values are mean (SD) unless otherwise specified

	Pain education	Usual care
	n = 31	n = 29
Women, n	22	18
Age	66 (9)	66 (10)
BMI	33 (5)	33 (7)
Civil status, n		
Cohabiting	25	19
Living alone	6	10
Daily smoker, n	3	4
Alcohol **^a^**, n	5	2
Educational level, n		
No education	10	10
< 3 years	8	4
≥ 3 years	8	7
Other	5	8
Work status, n		
Employed	6	8
Sick leave	3	2
Retired	22	19
Pain medication in the past week		
Paracetamol, n	27	26
Ibuprofen, n	16	13
Morphine or opioids, n	10	2
Amount of opioids/day, mg	33 (32)	10 (0)
Postoperative, n		
Operated in knee or hip within 1 year	3	3
Complications within 30 days **^b^**	1	1
Rehabilitation **^c^**	16	14
Primary outcome		
VAS during activity	48 (18)	49 (21)
Secondary outcome		
VAS during rest	19 (14)	25 (17)
OKS	21 (7)	22 (6)
KOOS Pain (n = 58)	40 (12)	37 (17)
EQ5D (n = 55), median (range)	0.66	0.72
	(0.16–0.83)	(–0.14 to 0.83)
PSEQ	33 (10)	34 (8)
PCS, median (range)	28 (23–48)	29 (23–52)
6-minute walk test	387 (106)	334 (103)
STS	10.3 (3.2)	9.2 (2.8)

a Consumption above recommendations (more than 1 unit of alcohol per day for women and 2 for men).

b Infection or brisement forcй.

c Community-based after the operation.

VAS, Visual Analog Scale; OKS, Oxford Knee Score;

KOOS, Knee Injury and Osteoarthritis Outcome Score;

EQ5D, EuroQol-5D; PSEQ, Pain Self-Efficacy Questionnaire;

PCS, Pain Catastrophizing Scale; STS, Sit-to-stand in 30 seconds.

### Outcome measures

**Figure 2.**
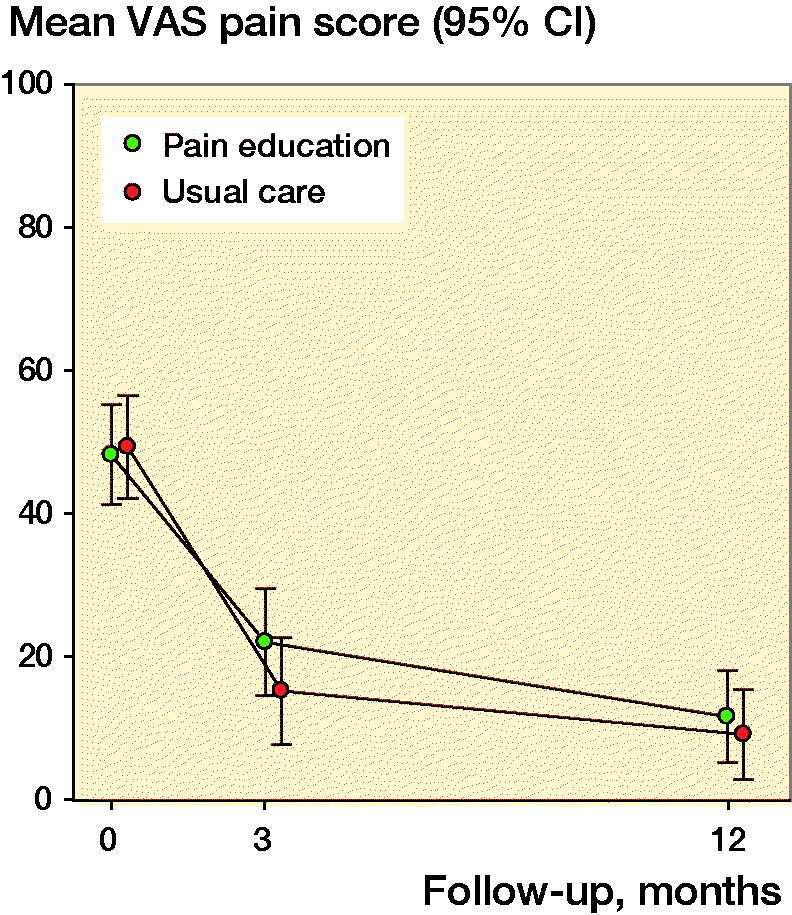
Pain during activity for the two groups over the study period. Mean Visual Analog Scale (VAS) scores with 95% CI in error bars for the pain education group (green dot) and the usual care group (red dot).

We found a mean difference of 3 (CI –6 to 11) mm in the primary outcome measure VAS during activity 12 months after TKA between the pain education group and the usual care group (p = 0.6). Mean VAS during activity 12 months postoperatively was 12 mm (CI 5–18) for the pain education group and 9 mm (CI 3–15) for the usual care group (Table 2). Both groups had statistically significant reductions in VAS from baseline to 12 months postoperatively. The pain education group reduced their VAS score during activity by a mean of 37 mm (CI 27–46) and the control group by a mean of 40 mm (CI 31–49) (Figure 2). The estimated group difference in change in VAS during activity from baseline to 12 months postoperatively was a mean of 3 (CI –10 to 16) mm. This difference was not statistically significant, and the CI does not include a clinically relevant difference between the 2 groups.

**Table 2.** Primary and secondary outcomes at baseline, 3 and 12 months. The estimates are given as mean values with 95% CI from the linear mixed effect model

	Pain education		Usual care	
	n = 31	n	n = 29	n
Primary outcome				
VAS during activity				
Baseline	48 (41–55)	31	49 (42–57)	29
3 months	22 (14–30)	24	15 (8–23)	24
12 months	12 (5–18)	24	9 (3–15)	26
Secondary outcome				
VAS during rest				
Baseline	19 (13–24)	31	25 (19–30)	29
3 months	11 (6–16)	24	7 (3–12)	25
12 months	7 (1–12)	24	6 (1–12)	26
Oxford Knee Score				
Baseline	21 (19–23)	31	22 (20–24)	29
3 months	28 (25–32)	24	31 (27–34)	25
12 months	33 (29–37)	24	37 (33–41)	24
KOOS pain				
Baseline	40 (35–45)	31	37 (32–43)	27
3 months	64 (57–72)	25	69 (61–77)	22
12 months	75 (67–82)	24	83 (75– 90)	23
EQ-5D				
Baseline	0.58 (0.52–0.66)	29	0.62 (0.54–0.70)	26
3 months **^a^**	0.72 (0.65–0.79)	24	0.82 (0.78–0.87)	23
12 months	0.78 (0.70–0.86)	24	0.86 (0.81–0.91)	24
PSEQ				
Baseline	33 (30–36)	31	34 (31–38)	29
3 months	43 (38–47)	25	47 (43–52)	25
12 months	49 (44–53)	23	52 (48–57)	25
PCS				
Baseline	30 (28–32)	31	31 (29–33)	29
3 months	13 (9–17)	25	11 (7–15)	25
12 months	11 (7–16)	23	9 (5–14)	25
6-minute walk test				
Baseline	387 (350–424)	31	334 (296–372)	29
3 months	405 (372–438)	24	375 (342–408)	25
12 months	441 (402–480)	24	406 (367–446)	26
Sit to stand				
Baseline	10 (9–11)	31	9 (8–10)	29
3 months	11 (9–13)	24	10 (8–11)	25
12 months	12 (11–14)	24	11 (10–13)	26

For abbreviations, see Table 1.

a p = 0.01

We found no statistically significantly differences between the 2 groups in any of the secondary outcomes (Table 2). In both groups there was a significant effect of time on OKS and PCS. The mean OKS score in the pain education group and the control group increased by a mean of 13 points (CI 9–16) and by a mean of 15 points (CI 11–18), respectively. The PCS score reduced by a mean 19 (CI 14–24) in the pain education group and by a mean of 22 (CI 17–26) in the control group.

## Discussion

Participation in patient education in addition to usual care for patients with a high pain catastrophizing score did not result in better outcomes 12 months after TKA. However, for both groups OKS and VAS during activity improved statistically significantly over time indicating that knee function improves and knee pain reduces after a TKA.

Recent research on the effect of CBT on pain severity has shown mixed results and meta analyses show that the effect size on pain is small (Dixon et al. 2007, Williams et al. 2012). Some studies report substantial improvements in patients with knee osteoarthritis (Somers et al. 2012, Cai et al. 2018) while others report no effect on pain when comparing a CBT group with a usual care group (Helminen et al. 2015, Broderick et al. 2016, Allen et al. 2019, Riddle et al. 2019).

We found no additive effect of pain education based on CBT on pain, physical function, quality of life, self-efficacy, or pain catastrophizing. There may be several reasons for this. 1st, most of the former studies reporting an effect of a CBT intervention on these factors have included patients with knee OA and only a few studies have included patients scheduled for TKA. Cai et al. (2018) found that a CBT program was superior to standard care in reducing kinesiophobia, pain catastrophizing, and knee pain among patients with high levels of kinesiophobia before a TKA. Contrary to this result, Riddle et al. (2019) found that an internet-based pain coping skills program for patients with high levels of pain catastrophizing before TKA did not improve pain or functional outcomes more than usual care. Their conclusion is consistent with that in our study, even though there are differences in the designs. We have a different primary outcome and cut-off regarding PCS. Further, the study from Riddle et al. tested telephone-based pain coping skills training contrary to our study where the patients came to the hospital and met a physiotherapist in person at all sessions. Patients scheduled for an operation may rely on the results of the operation to a great extent, which may result in less motivation for working with cognitive behavioral therapy. These expectations are not the same among patients with chronic knee OA not scheduled for an operation. Research has shown that patients’ baseline expectations regarding the benefit of CBT-based pain coping are associated with the effect of the program, and patients with lower expectation experience only a little benefit from the program (Goossens et al. 2005, Broderick et al. 2016).

2nd, based on existing research suggesting that pain catastrophizing is a predictor of persistent pain after a TKA, we decided to use PCS as a screening tool for inclusion of patients. However, recent research has suggested that patients with elevated scores on more than 1 risk factor are more likely to develop prolonged pain and disability, and that multiple psychological factors need to be considered with respect to pain and physical disability in knee OA (Sinikallio et al. 2014). Further research in this area is needed where pain catastrophizing is not the sole screening tool.

We wanted to include the one-third of our TKA population with the highest PCS score. Based on data collected in our department in the years 2011–2013 we defined a cut-off for inclusion at PCS > 22 in this study (Birch et al. 2019). The PCS user manual define patients with a PCS > 30 to be at high risk of developing chronic pain. This means that we have included patients with both moderate and high levels of pain catastrophizing. However, an additional analysis showed no correlation between preoperative PCS level and treatment effect (VAS during activity) (tested with Spearman’s rank correlation r = 0.18, p =0.4). Only limited research is available on pain catastrophizing cut-off scores for specific groups of patients.

Our intervention consisted of only CBT-based pain education and consistent with the study of Riddle et al. (2019) we found no statistically significant effect of this intervention compared with usual care on any of the outcomes. However, 2 studies have found that CBT-based pain coping combined with either behavioral weight management or exercise demonstrated statistically significantly better outcomes in terms of pain and function compared with PCST (Somers et al. 2012, Bennell et al. 2016). Combining psychological treatment with exercise is in line with the biopsychological approach to chronic pain management and future research may investigate the potential in combining CBT-based pain education with exercise.

For the psychological outcomes we expected a larger reduction in PCS and an increase in PSEQ among the patients in the intervention group compared with the usual care group because these issues were emphasized in the pain education. However, this was not the case and both groups achieved similar improvements after 12 months. 3 months after the operation both groups have a PCS score far under 21 points, which is the cuff point for moderate PCS.

### Limitations

There are some limitations too. 1st we had to change the number of sessions from 7 to 6 approximately 1 year after we started including patients. This means that not all patients have received the same number of sessions, but they all received the same content and we found no difference in the primary outcome between patients who received 7 sessions and patients who received 6 sessions. Furthermore, because we wanted to design a method of patient education that fits into a hospital setting, it consisted of only 7 sessions contrary to previous studies where the CBT intervention ranged from 10 to 12 sessions (Keefe et al. 2004). This might have affected the results. 2nd is the lack of blinding to the treatment groups, which was not possible. 3rd, the rather long inclusion period at about 2.5 years. However, there was no change in practice or surgeons during this period, so we do not think this has influenced the results. 4th, only patients with moderate to high pain catastrophizing score were studied and the results of this study cannot be generalized to other populations.

### Conclusion

This study showed that pain education based on CBT was not superior to usual care after TKA in terms of reducing pain or improving physical function, quality of life, self-efficacy, and pain catastrophizing. Future research is warranted to identify predictors of persistent pain and interventions for the approximately 20% of patients with persisting pain after a TKA.

The authors would like to thank all the participating patients, nurses, physiotherapists, and secretaries at the University Clinic for Hand, Hip, and Knee surgery and the Department of Physiotherapy and Occupational Therapy, Holstebro Regional Hospital, Hospital Unit West, Denmark.

SB, MS, IM, and TBH participated in the conception and design of the study and helped to revise the manuscript. All authors read and approved the final manuscript.

*Acta* thanks Johan Creutzfeldt and Kristian Kjaer Petersen for help with peer review of this study.
